# Diabetic and Hypertensive Retinopathy Screening in Fundus Images Using Artificially Intelligent Shallow Architectures

**DOI:** 10.3390/jpm12010007

**Published:** 2021-12-23

**Authors:** Muhammad Arsalan, Adnan Haider, Jiho Choi, Kang Ryoung Park

**Affiliations:** Division of Electronics and Electrical Engineering, Dongguk University, 30 Pildong-ro, 1-gil, Jung-gu, Seoul 04620, Korea; arsal@dongguk.edu (M.A.); adnanhaider@dgu.ac.kr (A.H.); choijh1027@dongguk.edu (J.C.)

**Keywords:** fundus images, diabetic retinopathy, hypertensive retinopathy, retinal disease screening, retinal vasculature, ophthalmic diseases

## Abstract

Retinal blood vessels are considered valuable biomarkers for the detection of diabetic retinopathy, hypertensive retinopathy, and other retinal disorders. Ophthalmologists analyze retinal vasculature by manual segmentation, which is a tedious task. Numerous studies have focused on automatic retinal vasculature segmentation using different methods for ophthalmic disease analysis. However, most of these methods are computationally expensive and lack robustness. This paper proposes two new shallow deep learning architectures: dual-stream fusion network (DSF-Net) and dual-stream aggregation network (DSA-Net) to accurately detect retinal vasculature. The proposed method uses semantic segmentation in raw color fundus images for the screening of diabetic and hypertensive retinopathies. The proposed method’s performance is assessed using three publicly available fundus image datasets: Digital Retinal Images for Vessel Extraction (DRIVE), Structured Analysis of Retina (STARE), and Children Heart Health Study in England Database (CHASE-DB1). The experimental results revealed that the proposed method provided superior segmentation performance with accuracy (Acc), sensitivity (SE), specificity (SP), and area under the curve (AUC) of 96.93%, 82.68%, 98.30%, and 98.42% for DRIVE, 97.25%, 82.22%, 98.38%, and 98.15% for CHASE-DB1, and 97.00%, 86.07%, 98.00%, and 98.65% for STARE datasets, respectively. The experimental results also show that the proposed DSA-Net provides higher SE compared to the existing approaches. It means that the proposed method detected the minor vessels and provided the least false negatives, which is extremely important for diagnosis. The proposed method provides an automatic and accurate segmentation mask that can be used to highlight the vessel pixels. This detected vasculature can be utilized to compute the ratio between the vessel and the non-vessel pixels and distinguish between diabetic and hypertensive retinopathies, and morphology can be analyzed for related retinal disorders.

## 1. Introduction

Medical image assessment is a vital tool for the computer-aided diagnosis of numerous diseases. Unlike conventional image processing tools, artificial intelligence algorithms based on deep learning are more popular in disease analysis owing to their reliability and versatility [1]. Several retinal diseases are associated with blindness or vision loss, and computer vision has a strong potential to analyze these retinal diseases by image analysis for early detection, diagnosis, and treatment [2]. Deep learning-based methods are extensively recognized for faster screening and computer-aided analysis of several retinal disorders associated with retinal anatomy [3]. Retinal blood vessels have an extremely complex structure and are important biomarkers for detecting and analyzing different retinal dysfunctions and other diseases [4,5]. As one of several deep learning schemes, medical image segmentation supports ophthalmologists and other medical doctors in numerous complex diagnoses. Segmentation schemes reduce the manual analysis of images for disease or symptom screening; semantic segmentation using deep learning is an advanced technique for pixel-wise medical image classification [6]. Diabetes and hypertension involve a change in the retinal vessels because of the abnormal growth or degeneration of these vessels, which can be detected by accurate retinal blood vessel segmentation [7]. The manual analysis and detection of this retinal vasculature is a tiresome and time-consuming task placing a diagnostic burden on medical specialists; clearly, automated methods could allow faster diagnoses [8]. Numerous diagnostic facets can be deduced from the thickness, tortuosity, creation, and elimination of these retinal vessels [9]. Diabetic retinopathy is a complication created by diabetes [10] where the retinal vessels can swell or leak fluid or blood owing to high blood sugar levels, which can lead to vision loss depending on the severity of the disease [11]. Hypertensive retinopathy is a retinal disease associated with hypertension that worsens with high blood pressure. Hypertensive retinopathy is associated with hypertension and causes narrowing of the retinal vessels, in particular, arteriolar and focal arteriolar narrowing [12]. Vascular changes related to hypertension and diabetes can be minute, requiring a keen observational analysis to perceive the relevant changes associated with the disease [13,14]. Deep learning has the potential to detect different diseases and provide diagnostic aid to medical specialists in different medical applications [15,16,17,18,19,20,21]. Similarly, deep learning-based semantic segmentation with robust architecture can detect minor changes in the retinal vasculature and aid medical specialists in observing the related changes for faster and early diagnoses. There are several deep feature-based schemes for retinal blood vessel detection [22]; however, there is a requirement for the development of low-cost robust methods that can accurately detect vasculature with minor changes. Moreover, existing segmentation-based methods [11,22,23] lack a complete solution for the detection of diabetic and hypertensive retinopathy; rather, these methods only focus on segmentation. To address these concerns, this study proposes two separate deep learning-based semantic segmentation architectures: dual-stream fusion network (DSF-Net) and dual-stream aggregation network (DSA-Net) for accurate retinal vasculature detection in fundus images. Both proposed networks are based on dual stream-based fusion and aggregation that combines the fine spatial information from both paths using feature fusion (for DSF-Net) and feature concatenation (for DSA-Net). The unique architecture of both networks allows them to provide accurate segmentation using a shallow architecture with minimal layers. Moreover, the proposed networks can detect vessels from fundus images without using conventional image processing schemes for image enhancement. Experimental evaluation using three public datasets, i.e., Digital Retinal Images for Vessel Extraction (DRIVE), Structured Analysis of Retina (STARE), and Children Heart Health Study in England Database (CHASE-DB1), confirmed that the proposed DSF-Net and DSA-Net provide superior segmentation performance. This study indicates that the new method has the following novel advantages compared to existing deep feature-based methods:

- The proposed method performs automatic segmentation of retinal vasculature, providing the opportunity for ophthalmic analysis of diabetic and hypertensive retinopathy and tracking of vascular changes.
- The proposed method avoids intensive conventional image processing schemes for the preprocessing of fundus images, and two separate networks DSF-Net and DSA-Net are provided with feature fusion and concatenation that consume only 1.5 million trainable parameters.
- The Dice pixel classification layer effectively addresses the class imbalance between the vessel and the non-vessel pixels.
- The proposed trained models and codes are open for reuse and fair comparison [24].

## 2. Material and Methods

### 2.1. Datasets

The proposed DSF-Net and DSA-Net were evaluated using three publicly available fundus image datasets: DRIVE [25], STARE [26], and CHASE-DB1 [27]. No potential ethical issues existed, because the datasets are publicly available for retinal image analysis. To evaluate the algorithms and for supervised learning, expert annotations were provided with these datasets. Further details of these datasets are provided below. Except for the information reported in Section 2.1.1, Section 2.1.2, Section 2.1.3, additional information regarding patients and their diseases is not available from the providers of three databases of DRIVE [22], STARE [23], and CHASE-DB1 [24].

#### 2.1.1. DRIVE

**Figure 1:**
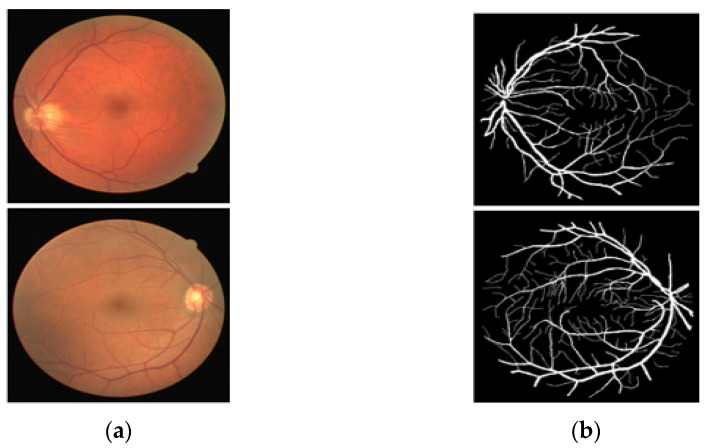
Example of images from DRIVE: (**a**) Original input image and (**b**) Expert annotation Mask.

The DRIVE [25] dataset is a publicly available fundus image dataset that includes 40 RGB fundus images collected from a retinal screening program in the Netherlands, part of the Utrecht database. These images are open to the public for retinal image analysis, and manually designed vessel masks by an expert are provided with the dataset for training and evaluation. The images are 565 × 584 pixels, captured by a fundus camera (Canon CR5 nonmydriatic 3CCD, New York, NY, USA) with a 45° field of view (FOV). The pathologies make the vessel appearance difficult to understand according to their proportion. In these 40 images, 7 images present a pathology (pigment epithelium changes, hemorrhages, exudates, etc.). Of these 7 images with pathology, 3 images are included in the train set, and the rest 4 images are included in the test set by the dataset providers. The manual vessel segmentation masks were carefully designed under the supervision of an ophthalmologist. Figure 1 presents sample images–annotation pairs, and Table 1 describes the train–test split details and cross-validation used in our experiments.

#### 2.1.2. STARE

**Figure 2:**
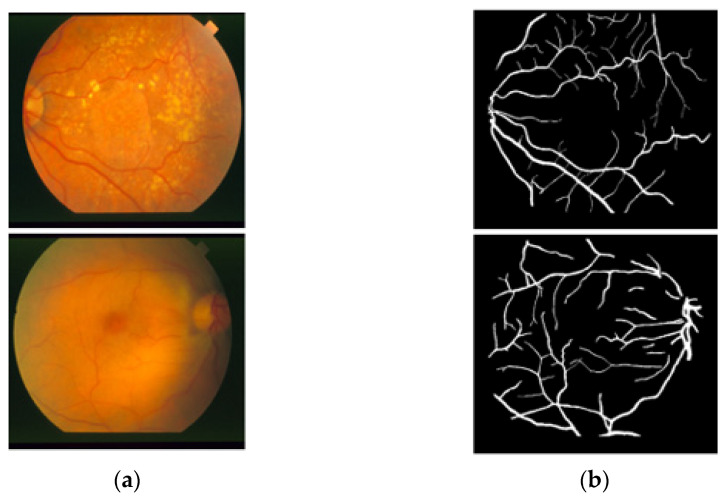
Example images from STARE: (**a**) Original input image and (**b**) Expert annotation mask.

The STARE [26] dataset includes 20 RGB fundus slide images−annotation pairs; the images were captured using a fundus camera (TopCon TRV-50, Tokyo, Japan). The dataset is publicly available for retinal image analysis, and manually designed vessel masks by an expert are provided with the dataset for training and evaluation. The images were taken with a 35° FOV and a size of 605 × 700 pixels. The description of the dataset states that vessel segmentation healthy fundus images are easy to evaluate compared to images with pathologies in which the vessel appearance is difficult to examine. Among these 20 images, 10 images present a pathology. Manual vessel segmentation masks were carefully designed by human observers, and the process of manual labeling took a long time due to the boundary pixels or the minor vessels pixels. Figure 2 presents sample images–annotation pairs, and Table 1 describes the train–test split details and cross-validation used in our experiments.

#### 2.1.3. CHASE-DB1

**Figure 3:**
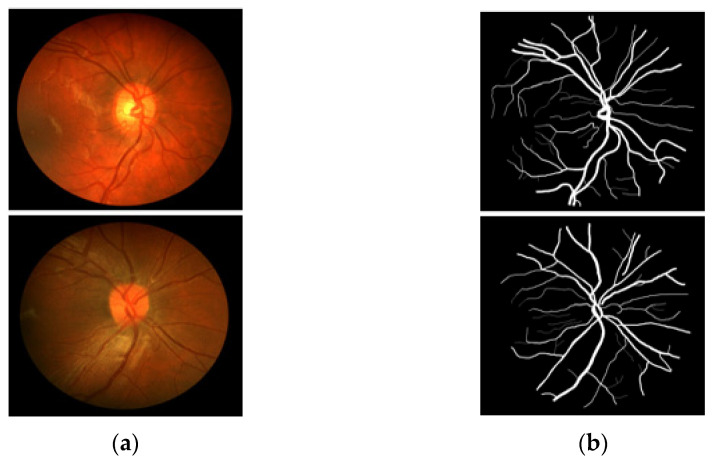
Example images from CHASE-DB1: (**a**) Original input image and (**b**) Expert annotation mask.

CHASE-DB1 [27] includes 28 RGB fundus images–annotation pairs acquired from multiethnic school children; the images are 960 × 999 pixels, captured using a fundus camera (Nidek NM-200D, Aichi, Japan) with a 30° FOV. This dataset was assembled as part of a cardiovascular health survey of 200 schools in London, Birmingham, and Leicester. The dataset is publicly available for retinal image analysis, and manually designed vessel masks by an expert are provided with the dataset for training and evaluation. The description of the dataset states that the dataset is characterized by poor contrast, non-uniform illumination, and central vessel reflex that make the segmentation difficult. Manual vessel segmentation masks were carefully designed by two independent human observers and were used for training and evaluation. Figure 3 presents sample images–annotation pairs, and Table 1 describes the train–test split details and cross-validation used in our experiments.

### 2.2. Method

#### 2.2.1. Summary of the Proposed Method

**Figure 4:**
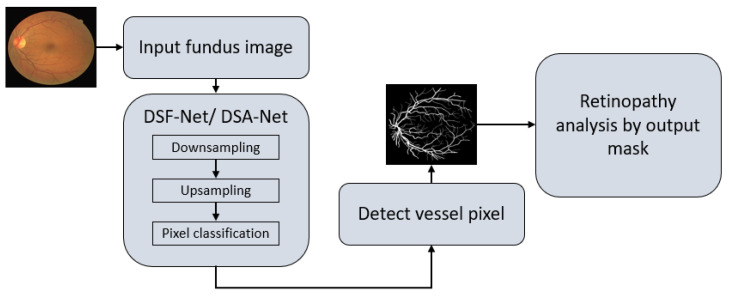
Overall summary of the proposed retinopathy analysis using DSF-Net or DSA-Net. Abbreviations: DSF-Net, dual stream fusion network, DSA-Net, dual stream aggregation network.

As explained in Section 1, vessel detection is very important for the assessment of several retinal diseases. Vessel detection from raw images is difficult due to non-uniform illumination. This study presents two deep learning-based segmentation architectures, DSF-Net and DSA-Net, for accurate retinal vasculature detection. Figure 4 presents a general summary of the proposed method. Each network (DSF-Net and DSA-Net) is a fully convolutional network that can segment the retinal vessels from the background without preprocessing of the input image. Multiple convolutions are applied in a feed–forward fashion for pixel-wise classification (semantic segmentation). Both networks provide a binary mask at the output with the presentation of the vessels and background pixels as “1” and “0”, respectively. The output mask from the proposed network has the representation of vessel pixels and non-vessel pixels and is then used for the analysis of diabetic and hypertensive retinopathy using V_r_, as explained in Section 4.1.

#### 2.2.2. Structure of the Proposed Method

**Figure 5:**
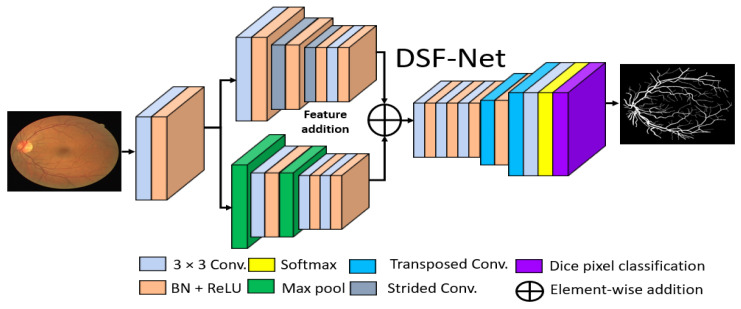
Proposed Dual-Stream Fusion Network (DSF-Net) Abbreviations: DSF-Net, dual stream fusion network, Conv., convolution, BN, batch normalization, ReLU, rectified linear unit.

**Figure 6:**
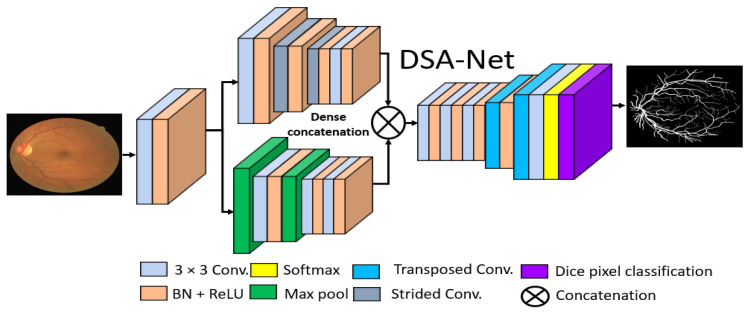
Proposed Dual-Stream Aggregation Network (DSA-Net). Abbreviations: DSA-Net, dual stream aggregation network, Conv., convolution, BN, batch normalization, ReLU, rectified linear unit.

The basic goal of this study was to produce an effective architecture that can detect retinal vessels accurately without using expensive image processing schemes for enhancement, with an improved design that consumes a low number of trainable parameters. To address these issues, this study presents two separate shallow networks, DSF-Net and DSA-Net, for retinal vessel segmentation, as displayed in Figure 5 and Figure 6, respectively. DSF-Net is based on residual feature fusion by the element addition of the two streams (indicated in Figure 5). DSA-Net is based on dense feature aggregation by depth-wise concatenation of the two streams (indicated in Figure 6). This structure is entirely different from conventional structures such as Segmentation Network (SegNet) [28] and U-Shaped network (U-Net) [29], which use the decoder in the same way as an encoder, obtaining a too deep architecture with a large number of trainable parameters. The Proposed DSF-Net and DSA-Net are based on dual-stream and use just two transposed convolution-based shallow decoders. The shallow architecture of DSF-Net and DSA-Net enable to exhibit 1.5 M trainable parameters; it can be noticed that SegNet [28] and U-Net [29] are with 29.46 M and 31.03 M trainable parameters, respectively.

#### 2.2.3. Encoder of the Proposed Architecture

Conventional semantic segmentation networks have a single-stream encoder which involves multiple pooling layers to reduce the feature map size inside the network, as the pooling layers have no trained weights, and it is a fact that the multiple pooling operation causes spatial information loss, which finally results in performance deterioration [30]. Considering this fact and our goal of vessel detection without preprocessing of the images, the proposed DSF-Net and DSA-Net use two stream-based feature flows inside the encoder. The first stream (the upper stream indicated in Figure 5 and Figure 6) of DSF-Net and DSA-Net does not use a pooling layer, as this causes spatial information loss. The second stream involves only two pooling layers, such that the first stream covers the spatial information loss by feature addition or dense concatenation.

We used two streams in our proposed method, one with pooling layers and the other without pooling layers. The pooling layers (without learned weights) highlight the significant features and reduce the spatial dimension of the feature map, but according to [30], multiple pooling causes spatial information loss that finally deteriorates the performance. The stream based on strided convolutions (with learned weights) can benefit from the combination with a pooling-based stream, which allows better segmentation performance.

**Figure 7:**
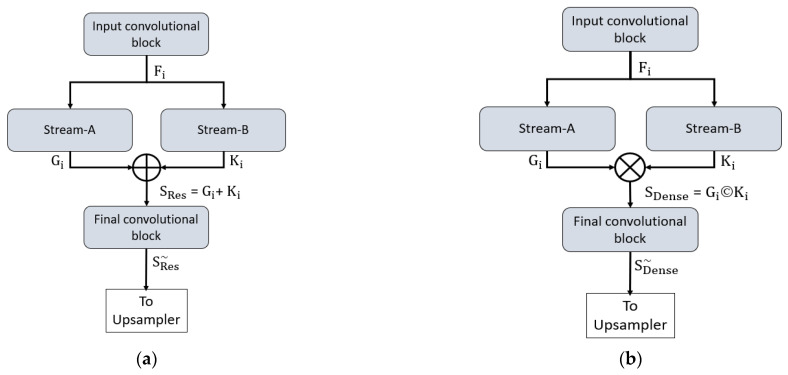
Connectivity pattern for the proposed feature combination: (**a**) Dual-stream feature fusion by element-wise addition in DSF-Net and (**b**) Dual-stream feature aggregation by depth-wise concatenation in DSA-Net.

Both proposed networks can retain the smallest feature map size, which is sufficiently large to avoid minor feature information loss and important for medical diagnosis applications. In detail, both networks maintain the final feature map at 163 × 163 pixels for a 650 × 650 image, which is sufficient to allow detection minor information. To understand the connectivity pattern inside DSF-Net and DSA-Net, Figure 7a,b provide mathematical and visual illustrations. In both networks, dual streams (Stream-A and Stream-B) are merged to create an enriched feature. According to Figure 7a, the input convolutional block provides the F_i_ feature to both streams, and Stream A and Stream B output the features G_i_ and K_i_, respectively. Note that the Stream-A features are without pooling, and the Stream-B features are with pooling. The features are fused residually to create a rich feature S_Res_, given by Equation (1).

(1) $$S_{\mathrm{Res}}=G_{i}+K_{i}$$

where “+” indicates the element-wise addition of features G_i_ and K_i_ from Stream-A and Stream-B. This S_Res_ feature is provided to the final convolutional block to create a feature $S_{\mathrm{Res}}^{\sim }$, which is the final feature from the DSF-Net for the upsampling operation to make the image size equal to that of the input image.

Similarly, based on Figure 7b, the input convolutional block provides the F_i_ feature to both streams, and Stream-A and Stream-B output the features G_i_ and K_i_, respectively. Note that the Stream-A features are without pooling, while the Stream-B features are with pooling. The features are aggregated by depth-wise dense concatenation to create the rich feature S_Dense_ given by Equation (2).

(2) $$S_{\mathrm{Dense}}=G_{i}{©\;K}_{i}$$

Here, © indicates the depth-wise concatenation of features G_i_ and K_i_ from Stream-A and Stream-B. This S_Dense_ feature is provided to the final convolutional block to create a feature $S_{\mathrm{Dense}}^{\sim }$, which is the final feature from the DSA-Net for the upsampling operation to make the image size equal to that of the input image. The DSF-Net and DSA-Net are two separate networks that respectively use element-wise addition and concatenation, as shown in Equations (1) and (2). The layer-wise activation map sizes are presented in Supplementary Materials Table S1a,b for DSF-Net and DSA-Net, respectively. It can be noticed from Supplementary Materials Table S1a that the feature map size from DSF-Net for both Stream-A and Stream-B results with feature map size 163 × 163 × 256 that remains identical after fusion. Furthermore, it can be noticed from Supplementary Materials Table S1b that map sizes from DSA-Net for both Stream-A and Stream-B are 163 × 163 × 256 and become 163 × 163 × 512 after concatenation; first convolution of final block (F-Conv-1) behaves as a bottleneck layer after the concatenation, reducing the number of channels in the upsampling part.

#### 2.2.4. Decoder of the Proposed Architecture

It can be observed from Figure 5 and Figure 6 that the upsampling task is performed by only two transposed convolutional layers, whereas conventional methods [28,29] also use the complete encoder-like structure for the decoder. The upsampling is used to attain a feature map size equal to the input size. Both the upper and the lower streams have a twofold smaller feature map size; these features from two streams are combined using element-wise addition or depth-wise concatenation. The merged feature of the encoder is provided to the decoder that uses the enriched feature maps for the upsampling operation. As this network provides a pixel-wise classification, the decoder uses the pixel classification layer at the end of the network that uses Generalized Dice Loss (GDL) for deep network training, as presented by Sudre et al. [31], where this loss function is associated with the generalized Dice score. This is an effective method to evaluate the segmentation performance with a single score in multiclass medical imaging problems [32].

#### 2.2.5. Experimental Environment and Data Augmentation

The proposed DFS-Net and DSA-Net were implemented on an Intel^®^ Core i7-3770K (Santa Clara, CA, USA) based desktop computer with Samsung 28 GB of RAM (Suwon, South Korea) using Microsoft Windows 10 (Washington, DC, USA) and MathWorks MATLAB R2021a (Massachusetts, USA). An NVIDIA GeForce GTX 1070 (Santa Clara, CA, USA) graphics processing unit was used in the experiments. The proposed models were trained from scratch without the use of any scheme for weight initialization, migration, sharing, or fine-tuning from other architectures. The important training hyperparameters are listed in Table 2. In deep networks, shallow architectures need more distinct data for successful training, and data augmentation is the scheme to increase the amount of training data. We used vertical flip, horizontal flip, X–Y translation to create 3840, 1344, and 1210 images from 20, 19, and 14 training images of DRIVE, STARE, and CHASE-DB-1, respectively. Supplementary Materials Figure S1a,b present the training accuracy and training loss curves for the DRIVE dataset, which indicate that the proposed architectures achieved greater training accuracy, with less training loss.

## 3. Results

### 3.1. Evaluation of the Proposed Method

The outputs of DSF-Net and DSA-Net are logical masks, with the vessel and non-vessel pixels represented by “1” and “0”, respectively. Following the versatile performance measure protocols [34], we assessed the performance of the proposed DSF-Net and DSA-Net using accuracy (Acc), sensitivity (SE), specificity (SP), and area under the curve (AUC) to fairly compare our method with existing state-of-the-art approaches. The mathematical formulations of Acc, SE, and SP are given by Equations (3)–(5), respectively.

(3) $$\mathrm{Acc}=\frac{\#\;\mathrm{of}\;\left(\mathrm{TP}+\mathrm{TN}\right)}{\#\;\mathrm{of}\;\left(\mathrm{TP}+\mathrm{FN}+\mathrm{FP}+\mathrm{TN}\right)}$$

(4) $$\mathrm{SE}=\frac{\#\;\mathrm{of}\;\left(\mathrm{TP}\right)}{\#\;\mathrm{of}\;\left(\mathrm{TP}+\mathrm{FN}\right)}$$

(5) $$\mathrm{SP}=\frac{\#\;\mathrm{of}\;\left(\mathrm{TN}\right)}{\#\;\mathrm{of}\;\left(\mathrm{TN}+\mathrm{FP}\right)}$$

### 3.2. Ablation Study

An ablation analysis was performed to explain the efficacy of DSA-Net in comparison with DSF-Net. Experiments proved that DSA-Net (with dense feature concatenation) outperformed DSF-Net (feature fusion by element-wise addition). In medical applications, false-negative pixels (judged by SE) are more important than false-positive pixels (judged by SP) [17]. DSA-Net provided superior SE compared to DSF-Net, maintaining the same number of trainable parameters and model size. It can be observed from Table 3 that feature concatenation resulted in a considerable performance difference.

### 3.3. Architectural and Visual Comparison of the Proposed Method with Existing Methods

This section presents a comparison of the proposed method with existing methods using Acc, SE, SP, and AUC mentioned in Section 3.1. Table 4 provides an architectural comparison (where architectural details are possible) between the proposed DSF-Net, DSA-Net and state-of-the-art architectures such as SegNet [28], U-Net [29], Vessel Segmentation Network (Vess-Net) [20], Attention Guided U-Net with Atrous Convolution (AA-UNet) [35], and Vessel Specific Skip Chain Convolutional Network (VSSC Net) [36]. The SegNet architecture is mainly used for multiclass road scene segmentation [28]; it was utilized by [37] for retinal blood vessel detection. Similarly, U-Net [29] is a widely accepted architecture proposed for medical image segmentation, which was used for retinal vessel segmentation [35] with the original U-Net and modified AA-UNet. Table 4 presents architectural and performance comparisons of the proposed networks and these widely accepted approaches. It can be observed from Table 4 (Acc, SE, SP) that the proposed DSF-Net and DSA-Net provided sufficiently comparable performance, with a reduced number of trainable parameters (1.5 M) and model size. Moreover, the proposed DSF-Net and DSA-Net required 81.25% fewer parameters compared to VSSC Net [36].

**Figure 8:**
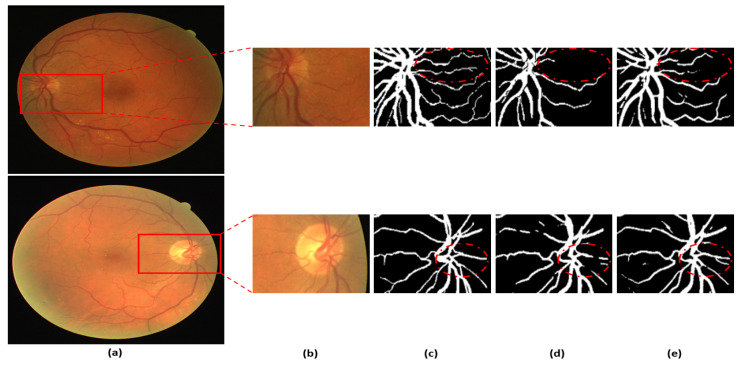
Visual comparison between DSA-Net, Vess-Net [20] (upper right), and U-Net [29] (lower right): (**a**) Original input image, (**b**) Enlarged input image, (**c**) Enlarged expert annotation, (**d**) Predicted mask image by Vess-Net [20] (upper), U-Net [29] (lower), and (**e**) Predicted image mask by DSA-Net.

Figure 8 presents a visual comparison of the proposed DSA-Net with Vess-Net [20] and U-Net [29]. As shown in o Figure 8, the vessels with low contrast compared to the background are not detected by Vess-Net [20] and U-Net [29], whereas DSA-Net, with dual-stream aggregation, lets the network utilize learned feature potential, which results in better segmentation performance. Moreover, DSA-Net is characterized by low pixel disruption of the segmented pixels compared to Vess-Net [20] and U-Net [29]. Supplementary Materials Section S1 and Table S2 present details of the statistical comparison based on two-tailed *t*-test [38] of the proposed DSA-Net with state-of-the-art approaches. Moreover, numerical comparisons of the proposed method with other methods are provided in Supplementary Materials Tables S3–S5 for DRIVE, STARE, and CHASE-DB1, respectively.

### 3.4. Visual Results of the Proposed Method for Vessel Segmentation

**Figure 9:**
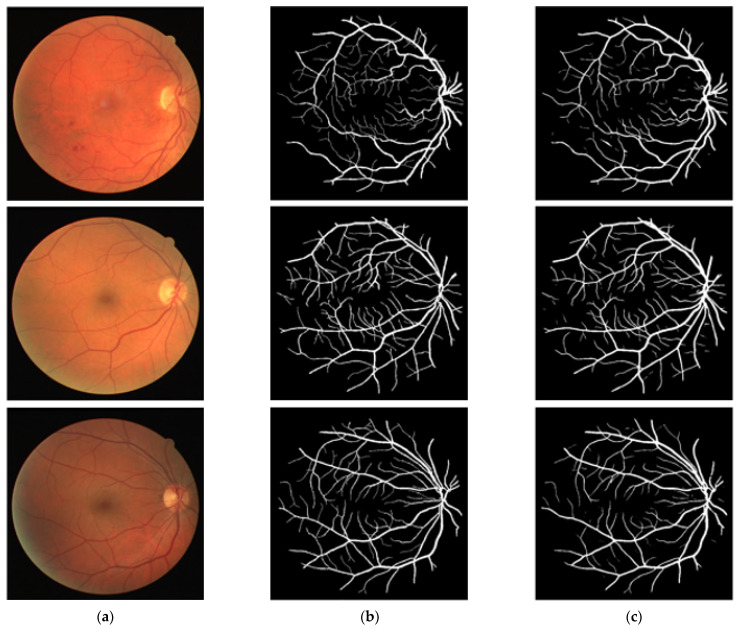
Visual results of the proposed DSA-Net Using the DRIVE Dataset: (**a**) Original input image, (**b**) Expert annotation, and (**c**) Predicted image mask by DSA-Net.

**Figure 10:**
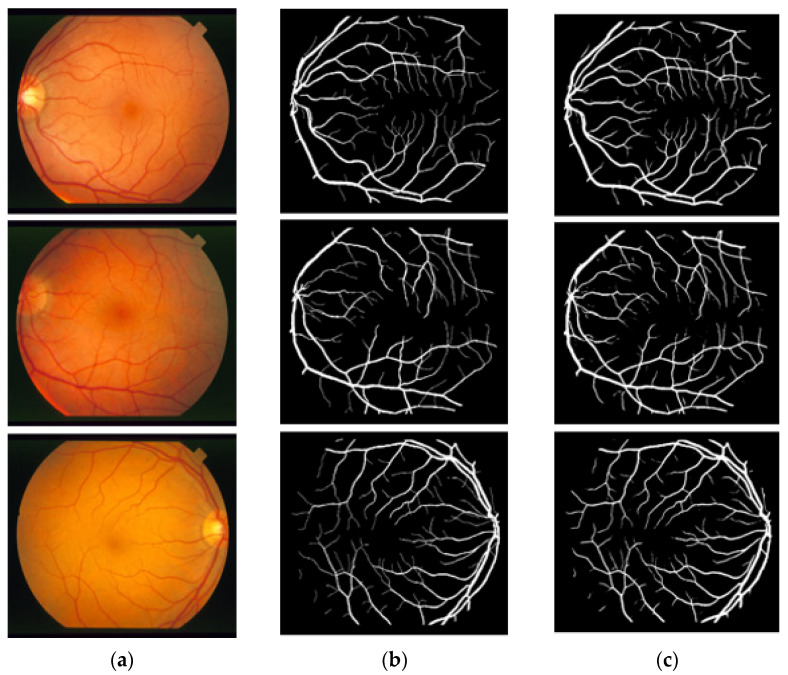
Visual results of the proposed DSA-Net using the STARE dataset: (**a**) Original input image, (**b**) Expert annotation, and (**c**) Predicted image mask by DSA-Net.

**Figure 11:**
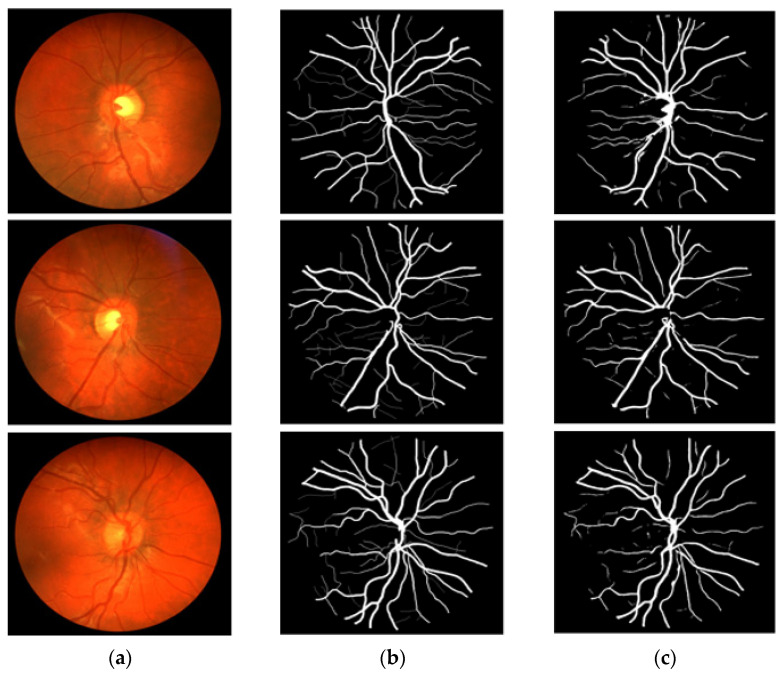
Visual results of the proposed DSA-Net using CHASE-DB1: (**a**) Original input image, (**b**) Expert annotation, and (**c**) Predicted image mask by DSA-Net.

In this section, the vessel segmentation visual results are presented for the publicly available DRIVE, STARE, and CHASE-DB1 retinal image datasets. The output of the network is a binary mask that is compared with an expert annotation mask for evaluation. Figure 9, Figure 10 and Figure 11 display the visual results of the proposed method; Figure 9, Figure 10 and Figure 11 display the (a) original input image, (b) expert annotation mask, and (c) predicted mask for the proposed method.

## 4. Discussion

Supplementary Materials Tables S3–S5 present a numerical result comparison of the proposed approach with existing methodologies for the DRIVE, STARE, and CHASE-DB1 datasets, respectively. It can be seen from Table 4 that the proposed DSF-Net and DSA-Net provide high Acc, SE, and SP, which is very important for medical diagnosis. The architecture comparison shows that both networks use the least number of trainable parameters and the smallest model size. According to Supplementary Materials Table S3, DSF-Net and DSA-Net provide the highest pixel accuracy of 96.93%. Sensitivity (SE) is very important in medical diagnosis, and DSA-Net showed the highest sensitivity of 82.68% for the DRIVE dataset. Although the Extreme ML [39] (in Supplementary Materials Table S3) provided the highest SP, the SE was very low. Lv et al.’s AA-UNet [35] (Supplementary Materials Table S3) provided a little higher AUC, but the Acc and SE were low compared to the of the DSA-Net; in addition, their network consumes a large number of trainable parameters. According to Supplementary Materials Table S4, Vess-Net [20] presented the highest Acc of 97.26%, whereas the proposed DSA-Net achieved the second-best Acc of 97.25% for the CHASE-DB1 dataset; the SE and AUC of the proposed method were higher than those of Vess-Net [20] which has a shallow architecture. Considering the STARE dataset, the proposed DSA-Net achieved the highest Acc of 97.00% and the highest SE of 86.07%, whereas the highest SP was achieved by Jin et al. [40], with lower Acc, SE, and AUC compared to the DSA-Net. It can be noticed from Supplementary Materials Tables S3–S5 that the proposed DSF-Net and DSA-Net provided a promising segmentation performance with a shallow low-cost architecture that requires a low number of parameters and a small model size. Gradient-weighted Class Activation Mapping (Grad-CAM) [41] displays the heat maps from a deep neural network representing the valuable features that are involved in predicting vessel class. Supplementary Materials Section S2 and Figure S2 report the Grad-CAM of different layers to validate the learning of the proposed method without bias. Further numerical comparison of proposed method with other methods [42,43,44,45,46,47,48,49,50,51,52,53,54,55,56,57,58,59] is available in Supplementary Tables S3–S5.

### 4.1. Principal Findings

The manual segmentation of the retinal vessel is a time-consuming process, and it is very difficult to understand vasculature changes because of their complex structure. The automatic segmentation of these retinal vessels can provide a better visual assessment for ophthalmic analysis. Recent advancements in deep learning have enabled developers to design semantic segmentation methods that can accurately detect retinal diseases in a complex scenario. The current computer-aided diagnosis based on semantic segmentation involves with complex and deep architectures that are inaccurate and consume many trainable parameters. Another problem of the existing approaches is that they use general image processing schemes for preprocessing and postprocessing, which increase the overall cost of the system. The existing methods just provide a segmentation mask, but no study describes how to use this mask as a base to detect retinal pathologies. In this study, two shallow semantic segmentation architectures are proposed to accurately segment the retinal vasculature without preprocessing. DSF-Net and DSA-Net provide accurate segmentation of retinal vessels by using raw images with only 1.5 million trainable parameters. Both networks (DSF-Net and DSA-Net) utilize dual-stream features (with and without pooling) that help detect minor vessels which are also important for early diagnosis. In medical diagnosis, the true positive rate (represented by sensitivity SE) is very important [17]; a high value of SE shows that a method has few false negatives. It can be noticed in Table 4 that DSA-Net showed the highest SE, which indicates that a dense connectivity helps to detect minor vessels.

The proposed network provides a binary mask as the output of the network (representing vessels pixel as “1” and background pixels as “0”). The output mask can provide the number of total pixels, number of vessel pixels, and number of non-vessel pixels available in the image. The V_r_ presented in Supplementary Materials Equation (A) is the ratio between the number of vessel pixels and that of background pixels, which can be computed with the predicted mask of the proposed method. Knowing that diabetic retinopathy causes inflammation of the retinal vessels (increased thickness) and hypertensive retinopathy causes shrinkage of the retinal vessels (decreased thickness) [20,60,61], this ratio can be utilized as a base to identify either diabetic or hypertensive retinopathy. This ratio can be compared in two consecutive visits to detect diabetic and hypertensive retinopathies (a greater V_r_ compared to previous visits representing diabetic retinopathy, a smaller V_r_ compared to previous visits representing hypertensive retinopathy). Supplementary Materials Figure S3a displays an exemplary image from the DRIVE dataset with a size of 565 × 584 pixels (total number of pixels, 329,960). Supplementary Materials Figure S3b presents a binary mask with 26,566 vessel pixels and 303,394 background pixels. For this image, V_r_ = 0.0876. The current V_r_ can be compared to the previous V_r_ for retinopathy analysis. Except for one previous study [20], no work has reported the screening procedure, providing only the segmentation mask. Even in [20], the authors considered only the number of vessel pixels as a biomarker to assess the retinal pathologies; however, the number of pixels can provide wrong results, as the image acquisition pattern changes. Regardless of the image acquisition conditions, the proposed method provides V_r_, which is the ratio between the vessel and the non-vessel pixel and can be an effective parameter, not much affected by image acquisition method and size.

Moreover, an accurate segmentation of the retinal vasculature provides the opportunity for ophthalmic analysis of retinal disorders that are related to vasculature morphology. Diabetic retinopathy, hypertensive retinopathy, retinal vein occlusion, and central retinal artery occlusion are examples of disorders that can be analyzed by the predicted mask using the proposed method. In addition, changes in retinal vessels can be analyzed by image subtraction, which provides precise information for disease diagnosis.

### 4.2. Limitations and Future Work

Although the proposed method provides superior segmentation performance, it has a few limitations. Medical images datasets usually contain a small amount of data that are insufficient to train a neural network. The datasets used in the current study contain a low number of images; therefore, data augmentation was used to synthetically generate images for better training of the proposed method. Similar to [20], the proposed method can be utilized to detect diabetic and hypertensive retinopathy using V_r_, which is based on accurate segmentation. Still, there is no direct publicly available dataset with expert segmentation masks that are specific for diabetic and hypertensive pathologies.

With a larger spatial size of the input images, shallower networks can usually perform well due to dense features available in the images. However, shallow architectures are prone to overfitting, which can be dealt with using different schemes. In the future, we intend to reduce the cost of the network by further decreasing the number of convolutions in a more efficient way. Moreover, in the future, we intend to prepare a fundus image dataset that is directly related to diabetic and hypertensive retinopathies at different stages, with annotation by expert ophthalmologists. In this way, it will be possible to evaluate the screening performance of future deep learning methods for these specific pathologies.

## 5. Conclusions

The main objective of this study was to create a framework that can be used to detect retinal vessels using raw images without preprocessing. This study presents DSF-Net and DSA-Net semantic segmentation architectures for the pixel-wise detection of retinal vessels. Dual-stream fusion and aggregation allow the network to better perform compared to classical approaches, without a preprocessing stage. The output of the proposed network is a binary mask that can be used to monitor the vasculature morphology and for the diagnosis and analysis of diabetic and hypertensive retinopathies. The optimum network design consumes only 1.5 million trainable parameters, with sufficiently acceptable segmentation performance. The proposed DSA-Net provides a greater true-positive rate compared to DSF-Net, which can be used to detect vascular changes in two successive visits. The proposed networks are sufficiently robust to provide accurate vessel detection, which can be used to support computer-aided ophthalmic diagnoses.

## Figures and Tables

**Table 1:** Description of DRIVE, STARE, and CHASE-DB1 Datasets and Experimentation Details. (The mentioned numbers of training and testing images refer to the original datasets, where data augmentation (described in Section 2.2.5) is used to increase the number of training images).

Database	Total Images	Test	Train	Image with Pathology	Cross-Validation	No. of Experiments
DRIVE [25]	40	20	20	7	Train–test defined	1
STARE [26]	20	1	19	10	Leave-one-out	20
CHASE-DB1 [27]	28	14	14	-	Two-fold	2

DRIVE: Retinal Images for Vessel Extraction, STARE: Structured Analysis of Retina, CHASE: Children Heart Health Study in England Database. The symbol “-“ shows that this information is not available in the reference study.

**Table 2:** Training hyperparameters used to train the proposed DFS-Net and DSA-Net.

Hyperparameters	Value
Initial learning rate	0.0001
Optimizer	Adam [33]
Epsilon	0.000001
Normalization	Global L2 normalization
Epochs	35
Iterations	11,200
Shuffling images	Each epoch

**Table 3:** Ablation study of the proposed DSF-Net and DSA-Net.

Method	Acc	SE	SP	AUC	Parameters	Model Size
DSF-Net (Proposed)	**96.93**	81.94	**98.38**	98.30	**1.5 M**	**3.63 MB**
DSA-Net (Proposed)	**96.93**	**82.68**	98.30	**98.42**	**1.5 M**	3.81 MB

Abbreviations: DSF-Net, dual stream fusion network, DSA-Net, dual stream aggregation network, Acc, accuracy, SE, sensitivity, SP, specificity. Statistically significant values are marked with Bold.

**Table 4:** Architecture and model comparison of the proposed DSF-Net and DSA-Net with current state-of-the-art schemes for the DRIVE dataset.

Method	Acc	SE	SP	No. of 3 × 3 Convolutions	No. of Parameters (million)	Model Size
Vess-Net [20]	96.55	80.22	98.10	16	9.7	36.6 MB
U-Net [29] **	96.78	81.34	98.27	18	31.03	70.9 MB
U-Net [35]	95.54	78.49	98.02	18	31.03	-
AA-UNet [35]	95.58	79.41	97.98	16	28.25	-
VSSC Net [36]	96.27	78.27	98.21	-	8	-
SegNet [37]	94.8	74.6	91.7	26	29.46	-
DSF-Net (Proposed)	**96.93**	81.94	**98.38**	9	**1.5**	**3.63 MB**
DSA-Net (Proposed)	**96.93**	**82.68**	98.30	9	**1.5**	3.81 MB

Abbreviations: DSF-Net, dual stream fusion network, DSA-Net, dual stream aggregation network, Acc, accuracy, SE, sensitivity, SP, specificity, AUC, area under the curve. Statistically significant values are marked with Bold, and “-” show that this value is not available in respective study. ** U-Net [35] code is not publicly available; therefore, we trained and tested U-Net [29] using the same criteria as those of the proposed method.

## Data Availability

Not applicable.

## Supplementary Materials

The following supporting information can be downloaded at: https://www.mdpi.com/article/10.3390/jpm12010007/s1, Table S1 (a): Feature map size elaboration of DSF-Net; Table S1 (b): Feature map size elaboration of DSA-Net; Figure S1: Training Accuracy and Loss Curves of (a) DSF-Net and (b) DSA-Net Training on DRIVE Dataset; Section S1: Statistical Comparison of the Proposed Method with State-of-the-Art Methods; Table S2: Statistical *t*-test analysis using *p*-value and confidence score for DSA-Net in comparison with U-Net [53] and Vess-Net [20]; Table S3: Numerical results comparison of the proposed DSF-Net and DSA-Net with existing approaches for the DRIVE dataset; Table S4: Numerical results comparison of the proposed DSA-Net with existing approaches for the STARE dataset; Table S5: Numerical results comparison of the proposed DSA-Net with existing approaches for the CHASE-DB1 dataset; Section S2: Grad-CAM Explanation of the Proposed Method; Figure S2: Grad-Cam heat maps for three sample images from the DRIVE, STARE, and CHASE-DB1 datasets (first, second, and third rows, respectively), with (a) Original image, (b) Expert annotation mask, Grad-CAM from (c) F-Conv-3, (d) F-TConv-1, (e) F-TConv-2, and (f) F-Conv-3 of Table S1 (Supplementary Materials); Figure S3: Sample Images from the DRIVE dataset: (a) Original Image and (b) predicted mask Image by the proposed method; Equation (A).
