# Supplementary material for: Diabetic and Hypertensive Retinopathy Screening in Fundus Images Using Artificially Intelligent Shallow Architectures
Source: J Pers Med. 2021 Dec 23;12(1):7. doi: 10.3390/jpm12010007 (PMC8777982; doi:10.3390/jpm12010007)
Supplement: Supplementary file 1 [file jpm-12-00007-s001.zip › jpm-1512735-supplementary.pdf]

Journal of
*Personalized*
*Medicine*

MDPI

# Supplementary Materials

**Table S1. (a).** Feature Map Size Elaboration for DSF-Net.

| Block/Stream | Layer/Size (Stride) | Filters | Output | No. of Parameters |
|---|---|---|---|---|
| Input Block | In-Conv/ 1 × 1 × 3 (S = 1) | 32 | 650 × 650 × 32 | 128 + 64 |
| Stream-A | A-Conv-1/ 3 × 3 × 32 (S = 1) | 32 | 650 × 650 × 32 | 9248 + 64 |
| | A-Conv-2/ 3 × 3 × 32 (S = 2) | 64 | 325 × 325 × 64 | 18,496 + 128 |
| | A-Conv-3/ 3 × 3 × 64 (S = 2) | 128 | 163 × 163 × 128 | 73,856 +256 |
| | A-Conv-3/ 3 × 3 × 128 (S = 1) | 256 | 163 × 163 × 256 | 295,168 + 512 |
| Stream-B | Max-Pool-1 (2 × 2) (S = 2) | - | 325 × 325 × 32 | - |
| | B-Conv-1/ 3 × 3 × 32 (S = 1) | 64 | 325 × 325 × 64 | 18,496 + 128 |
| | Max-Pool-2 (2 × 2) (S = 2) | - | 163 × 163 × 64 | - |
| | B-Conv-2/ 3 × 3 × 64 (S = 1) | 128 | 163 × 163 × 128 | 73,856 + 256 |
| | B-Conv-3/ 3 × 3 × 128 (S = 1) | 256 | 163 × 163 × 256 | 295,168 + 512 |
| Addition | Stream-A + Stream-B | - | 163 × 163 × 256 | - |
| Final block | F-Conv-1/ 1 × 1 × 256 (S = 1)/ F-Conv-1/ 1 × 1 × 512 (S = 1) | 256 | 163 × 163 × 256 | 131,328 + 512 |
| | F-Conv-2/ 3 ×3 × 256 (S = 1) | 128 | 163 × 163 × 128 | 259,040 + 256 |
| | F-Conv-3/ 3 × 3 × 128 (S = 1) | 64 | 163 × 163 × 64 | 73,792 + 128 |
| | F-TConv-1/ 2 × 2 × 64 (S = 2) | 32 | 325 × 325 × 32 | 8224 + 64 |
| | F-TConv-2/ 2 ×2 × 32 (S = 2) | 16 | 650 × 650 × 16 | 2064 + 32 |
| | F-Conv-4/ 1 × 1 × 16 (S = 1) | 2 | 650 × 650 × 2 | 34 + 4 |

Every convolution/transposed convolution has associated batch normalization (BN) and rectified linear unit (ReLU). Abbreviations: S = stride, In-Conv = input convolution, A-Conv = convolution for Stream-A, Max-Pool = max pooling, B-Conv = convolution for Stream-B, F-Conv = convolution for final block, and F-TConv = transposed convolution for final block. The symbol "-" show that this value is not available.

**Table S1. (b).** Feature Map Size Elaboration for DSA-Net.

| Block/Stream | Layer/Size (Stride) | Filters | Output | No. of Parameters |
|---|---|---|---|---|
| Input Block | In-Conv/ 1 × 1 × 3 (S = 1) | 32 | 650 × 650 × 32 | 128 + 64 |
| Stream-A | A-Conv-1/ 3 × 3 × 32 (S = 1) | 32 | 650 × 650 × 32 | 9248 + 64 |
| | A-Conv-2/ 3 × 3 × 32 (S = 2) | 64 | 325 × 325 × 64 | 18,496 + 128 |
| | A-Conv-3/ 3 × 3 × 64 (S = 2) | 128 | 163 × 163 × 128 | 73,856 +256 |
| | A-Conv-3/ 3 × 3 × 128 (S = 1) | 256 | 163 × 163 × 256 | 295,168 + 512 |
| Stream-B | Max-Pool-1 (2 × 2) (S = 2) | - | 325 × 325 × 32 | - |
| | B-Conv-1/ 3 × 3 × 32 (S = 1) | 64 | 325 × 325 × 64 | 18,496 + 128 |
| | Max-Pool-2 (2 × 2) (S = 2) | - | 163 × 163 × 64 | - |
| | B-Conv-2/ 3 × 3 × 64 (S = 1) | 128 | 163 × 163 × 128 | 73,856 + 256 |
| | B-Conv-3/ 3 × 3 × 128 (S = 1) | 256 | 163 × 163 × 256 | 295,168 + 512 |
| Concatenation | Stream-A © Stream-B | - | 163 × 163 × 512 | - |
| Final block | F-Conv-1/ 1 × 1 × 256 (S = 1)/ F-Conv-1/ 1 × 1 × 512 (S = 1) | 256 | 163 × 163 × 256 | 131,328 + 512 |
| | F-Conv-2/ 3 ×3 × 256 (S = 1) | 128 | 163 × 163 × 128 | 259,040 + 256 |
| | F-Conv-3/ 3 × 3 × 128 (S = 1) | 64 | 163 × 163 × 64 | 73,792 + 128 |
| | F-TConv-1/ 2 × 2 × 64 (S = 2) | 32 | 325 × 325 × 32 | 8224 + 64 |
| | F-TConv-2/ 2 ×2 × 32 (S = 2) | 16 | 650 × 650 × 16 | 2064 + 32 |
| | F-Conv-4/ 1 × 1 × 16 (S = 1) | 2 | 650 × 650 × 2 | 34 + 4 |

Every convolution/transposed convolution has associated batch normalization (BN) and rectified linear unit (ReLU). Abbreviations: S = stride, In-Conv = input convolution, A-Conv = convolution for Stream-A, Max-Pool = max pooling, B-Conv = convolution for Stream-B, F-Conv = convolution for final block, and F-TConv = transposed convolution for final block. The symbol "-" show that this value is not available. The symbol "©" indicates the depth-wise concatenation.

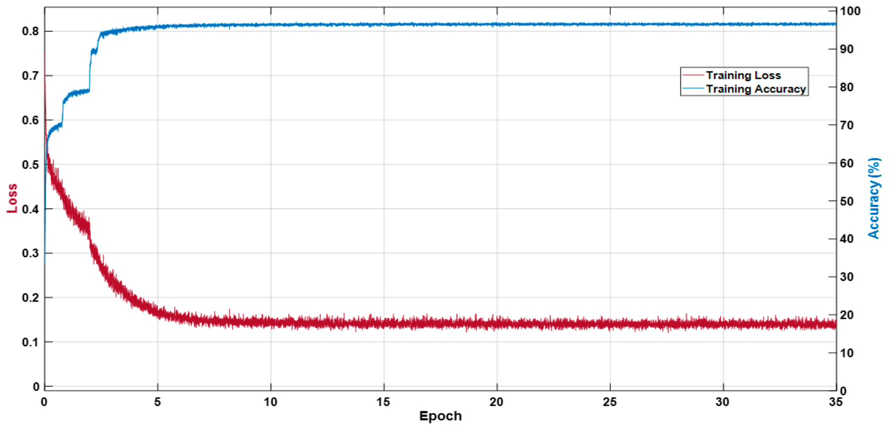


(**a**)

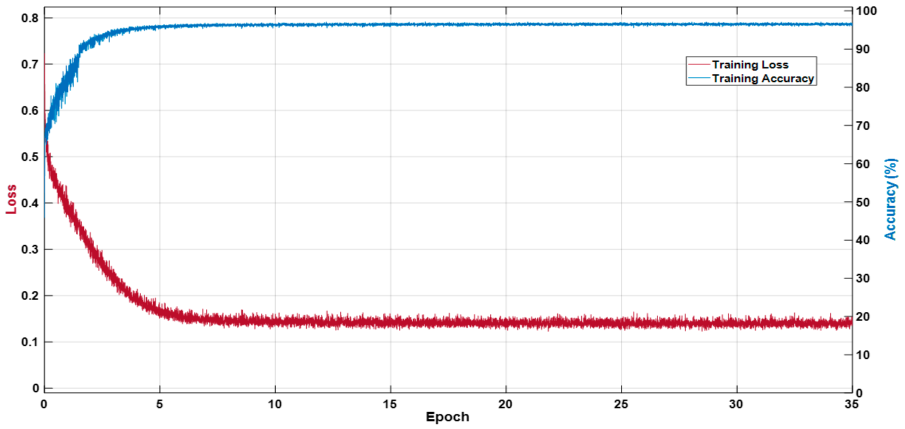


(**b**)

**Figure S1.** Training Accuracy and Loss Curves of (**a**) DSF-Net and (**b**) DSA-Net Training on DRIVE Dataset.

**Section. S1.** Statistical Comparison of the Proposed Method with State-of-the-Art Methods

Table S2 presents the statistical two-tailed *t*-test [38] to prove the efficacy of the proposed dual stream aggregation network (DSA-Net); a *t*-test is generally performed to highlight the performance difference between two methods with the null hypothesis (H). A hypothesis rejection score (*p*-value) was calculated with a confidence score for the rejection of the null hypothesis. In detail, the DSA-Net was statically compared with U-shaped network (U-Net ) [29] and vessel segmentation network (Vess-Net) [20], whose algorithms are publicly available for evaluation. According to Table S2, in comparison with U-Net [29], DSA-Net rejected the null hypothesis, with *p*-values of 0.02 and 0.032 and confidence scores of 98% and 96.8% for accuracy (Acc) and sensitivity (SE), respectively. It can also be noticed in Table S2 that, in comparison with Vess-Net [20], the proposed DSA-Net rejected the null hypothesis, with *p*-values of 0.03 and 0.085 and confidence scores of 97% and 91.5% for Acc and SE, respectively. This confirmed that the proposed DSA-Net is better than the second and third best state-of-the-art methods U-Net [29] and Vess-Net [20].

**Table S2.** Statistical *t*-test analysis using *p*-value and confidence score for DSA-Net in comparison with U-Net [53] and Vess-Net [20].

| ***t*-test** | **Acc** | | **SE** | |
|---|---|---|---|---|
| **Methods** | ***p*-Value** | **Confidence Score** | ***p*-Value** | **Confidence Score** |
| DSA-Net vs U-Net [29] | 0.02 | 98.0% | 0.032 | 96.8% |
| DSA-Net vs Vess-Net [20] | 0.03 | 97.0% | 0.085 | 91.5% |

Abbreviations: Acc, accuracy, SE, sensitivity, DSA-Net, dual stream aggregation network, U-Net, U-Shaped network, Vess-Net, vessel segmentation network.

**Table S3.** Numerical results comparison of the proposed DSF-Net and DSA-Net with existing approaches for the DRIVE dataset.

| **Method** | **Acc** | **SE** | **SP** | **AUC** |
|---|---|---|---|---|
| SegNet [37] | 94.8 | 74.6 | 91.7 | 26 |
| U-Net [35] | 95.54 | 78.49 | 98.02 | 18 |
| AA-UNet [35] | 95.58 | 79.41 | 97.98 | 16 |
| Vess-Net [20] | 96.55 | 80.22 | 98.10 | 16 |
| VSSC Net [36] | 96.27 | 78.27 | 98.21 | - |
| Zhang et al. [42] | 94.63 | 78.95 | 97.01 | - |
| Zhang et al. (postprocessing) [42] | 94.66 | 78.61 | 97.12 | - |
| 7-layered CNN [43] | - | 75.37 | 96.94 | - |
| Extreme ML [39] | 96.07 | 71.40 | **98.68** | - |
| Girard et al. Joint segment [44] | 95.7 | 78.4 | 98.1 | 97.2 |
| Hu et al. [45] | 95.33 | 77.72 | 97.93 | 97.59 |
| Fu et al. [46] | 95.23 | 76.03 | - | - |
| Cascaded CNN [47] | 95.41 | 76.48 | 98.17 | - |
| Soomro et al. [37] | 94.6 | 74.6 | 91.7 | 83.1 |
| DISCERN [48] | - | 78.81 | 97.41 | 96.46 |
| Yan et al. 3-stage DL [49] | 95.38 | 76.31 | 98.20 | 97.50 |
| Soomro et al. FCNN [50] | 94.8 | 73.9 | 95.6 | 84.4 |
| Jin et al. [40] | 95.66 | 79.63 | 98.00 | 98.02 |
| Leopold et al. [51] | 91.06 | 69.63 | 95.73 | 82.68 |
| Wang et al. [52] | 95.11 | 79.86 | 97.36 | 97.40 |
| Feng et al. [53] | 95.28 | 76.25 | 98.09 | 96.78 |
| Lv et al. U-Net [35] | 95.54 | 78.49 | 98.02 | 97.77 |
| Lv et al. AA-UNet [35] | 95.58 | 79.41 | 97.98 | **98.47** |
| Oliveira et al. [54] | 95.76 | 80.39 | 98.04 | 98.21 |
| Image BTS-DSN [55] | 95.51 | 78.00 | 98.06 | 97.96 |
| Patch BTS-DSN [55] | 95.61 | 78.91 | 98.04 | 98.06 |
| VessSeg [56] | 96.20 | 82.55 | 97.60 | 97.30 |
| Kromm et al. [57] | 95.47 | 76.51 | 98.18 | 97.50 |
| Li et al. [58] | 95.68 | 79.21 | 98.10 | 98.06 |
| DSF-Net (Proposed) | **96.93** | 81.94 | 98.38 | 98.30 |
| DSA-Net (Proposed) | **96.93** | **82.68** | 98.30 | 98.42 |

Abbreviations: DSF-Net, dual stream fusion network, DSA-Net, dual stream aggregation network, DRIVE, digital retinal images for vessel extraction, Acc, accuracy, SE, sensitivity, SP, specificity, AUC, area under the curve, SegNet, segmentation network, U-Net, U-Shaped network, AA-UNet, Attention Guided U-Net with Atrous Convolution, Vess-Net, vessel segmentation network, VSSC Net, vessel specific skip chain convolutional network, CNN, convolutional neural network, ML, machine learning, DISCERN, deep visual codebook framework for segmentation, DL, deep learning, FCNN, fully convolutional neural network, BTS-DSN, multi-scale, deeply supervised network with short connections, VessSeg, vessel segmentation. Statistically significant values are marked with Bold, and "-" show that this value is not available in respective study.

**Table S4.** Numerical results comparison of the proposed DSA-Net with existing approaches for the STARE dataset.

| Method | Acc | SE | SP | AUC |
|---|---|---|---|---|
| Zhang et al. [42] | 95.13 | 77.24 | 97.04 | - |
| Zhang et al. (postprocessing) [42] | 95.47 | 78.82 | 97.29 | - |
| Hu et al. [45] | 96.32 | 75.43 | 98.14 | 97.51 |
| Fu et al. [46] | 95.85 | 74.12 | - | - |
| Cascaded CNN [47] | 96.40 | 75.23 | 98.85 | - |
| Soomro et al. FCNN [37] | 94.8 | 74.8 | 92.2 | 83.5 |
| DISCERN [48] | - | 82.69 | 98.04 | 98.37 |
| CNN [59] | 96.17 | 78.23 | 97.70 | - |
| Q-CNN [59] | 95.87 | 77.92 | 97.40 | - |
| PQ-CNN [59] | 95.81 | 75.99 | 97.57 | - |
| Yan et al. [49] | 96.38 | 77.35 | 98.57 | 98.33 |
| Soomro et al. [50] | 94.7 | 74.8 | 96.2 | 85.5 |
| Jin et al. [40] | 96.41 | 75.95 | **98.78** | 98.32 |
| Leopold et al. [51] | 90.45 | 64.33 | 94.72 | 79.52 |
| Wang et al. [52] | 95.38 | 79.14 | 97.22 | 97.04 |
| Feng et al. [53] | 96.33 | 77.09 | 98.48 | 97.0 |
| Oliveira et al. [54] | 96.94 | 83.15 | 98.58 | **99.05** |
| Vess-Net [20] | 96.97 | 85.26 | 97.91 | 98.83 |
| Image BTS-DSN [55] | 96.60 | 82.01 | 98.28 | 98.72 |
| Patch BTS-DSN [55] | 96.74 | 82.12 | 98.43 | 98.59 |
| VessSeg [56] | 96.23 | 83.18 | 97.58 | 97.58 |
| Li et al. [58] | 96.78 | 83.52 | 98.23 | 98.75 |
| DSA-Net (Proposed) | **97.00** | **86.07** | 98.00 | 98.65 |

Abbreviations: DSA-Net, dual stream aggregation network, STARE, structured analysis of retina, Acc, accuracy, SE, sensitivity, SP, specificity, AUC, area under the curve, CNN, convolutional neural network, FCNN, fully convolutional neural network, DISCERN, deep visual codebook framework for segmentation, Q-CNN, quantized convolutional neural network, PQ-CNN, pruned quantized convolutional neural network, Vess-Net, vessel segmentation network, BTS-DSN, multi-scale, deeply supervised network with short connections, VessSeg, vessel segmentation. Statistically significant values are marked with Bold, and "-" show that this value is not available in respective study.

**Table S5.** Numerical results comparison of the proposed DSA-Net with existing approaches for the CHASE-DB1 dataset.

| Methods | Acc | SE | SP | AUC |
|---|---|---|---|---|
| Zhang et al. [42] | 94.97 | 77.86 | 96.94 | - |
| Zhang et al. (PP) [42] | 95.02 | 76.44 | 97.16 | - |
| Fu et al. [46] | 94.89 | 71.30 | - | - |
| Cascaded CNN [47] | 96.03 | 77.30 | 97.92 | - |
| Yan et al. 3-stage DL [49] | 96.07 | 76.41 | 98.06 | 97.76 |
| Jin et al. [40] | 96.10 | 81.55 | 97.52 | 98.04 |
| Leopold et al. [51] | 89.36 | **86.18** | 89.61 | 87.90 |
| Lv et al. U-Net [35] | 95.77 | 83.99 | 96.98 | 97.80 |
| Lv et al. AA-UNet [35] | 96.08 | 81.76 | 97.04 | **98.65** |
| Oliveira et al. [54] | 96.53 | 77.79 | **98.64** | 98.55 |
| Vess-Net [20] | **97.26** | 82.06 | 98.41 | 98.0 |
| Image BTS-DSN [55] | 96.27 | 78.88 | 98.01 | 98.40 |
| VessSeg [56] | 96.20 | 82.91 | 97.30 | 97.65 |
| Li et al. [58] | 96.35 | 78.18 | 98.19 | 98.10 |
| DSA-Net (Proposed) | 97.25 | 82.22 | 98.38 | 98.15 |

Abbreviations: DSA-Net, dual stream aggregation network, CHASE-DB1, and children heart health study in England database, Acc, accuracy, SE, sensitivity, SP, specificity, AUC, area under the curve, CNN, convolutional neural network, DL, deep learning, U-Net, U-Shaped network, AA-UNet, Attention Guided U-Net with Atrous Convolution, Vess-Net, vessel segmentation network, BTS-DSN, multi-scale, deeply supervised network with short connections, VessSeg, vessel segmentation. Statistically significant values are marked with Bold, and "-" show that this value is not available in respective study.

**Section. S2.** Grad-CAM Explanation of the Proposed Method

Gradient-weighted class activation mapping (Grad-CAM) [41] displays the heat maps from a deep neural network representing the valuable features that are involved in predicting a specific class. Grad-Cam displays feature maps that are averaged along the channels of the feature map, where red indicates a high confidence score, and blue represents evidence of the class. Figure S2 displays the Grad-CAM images for the Digital Retinal Images for Vessel Extraction (DRIVE), Structured Analysis of Retina (STARE), and Children Heart Health Study in England Database (CHASE-DB1) datasets, respectively. These feature maps were extracted from F-Conv-3, F-TConv-1, F-TConv-2, and F-TConv-3 (final block convolution-3, final block transposed convolution-1, final block transposed convolution-2, final block transposed convolution-3 ), as shown in Table S1. The learning of the proposed network is evident in Figure S2 without bias. The network was capable of distinguishing the vessel pixels from the background. The following are the conclusions of this study:

- The optimum architecture enabled the network to perform an acceptable segmentation without a preprocessing stage.
- Dual-stream features (with and without pooling) learned valuable features and reduced spatial loss.
- Dense aggregation assisted in feature empowerment and created a collective concatenated feature that allowed a faster convergence.
- Unlike conventional encoder–decoder architectures, it was not necessary to build a decoder that was the same as the encoder, and the parameters could be saved by implementing upsampling with minimal layers.
- Dense concatenation alleviated the feature latency problem; therefore, dense aggregation outperformed a network with element-wise feature addition. The final feature

map size of the downsampled image is important, and the segmentation performance can be improved if the features inside the network are not significantly downsized. The final feature map size in the proposed method was 163 × 163 for a 650 × 650, which was sufficiently large to represent the spatial information.

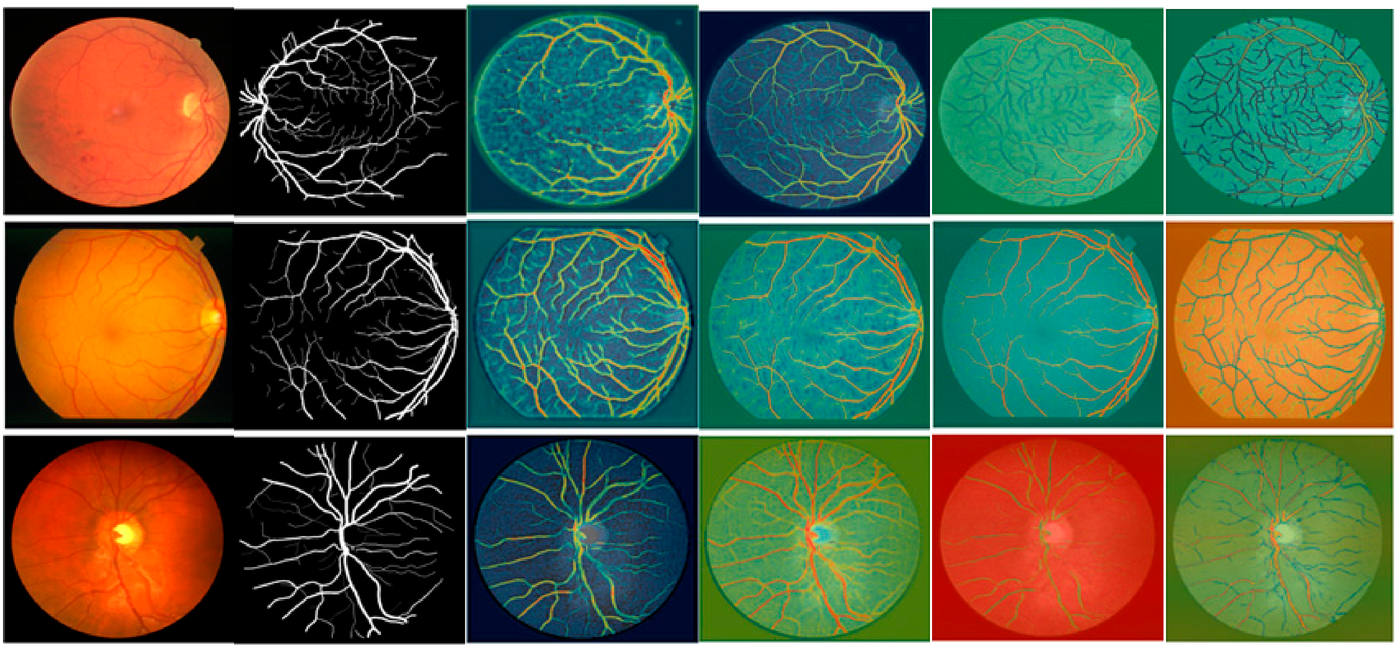


**Figure S2.** Grad-Cam heat maps for three sample images from the DRIVE, STARE, and CHASE-DB1 datasets (first, second, and third rows, respectively), with (**a**) Original image, (**b**) Expert annotation mask, Grad-CAM from (**c**) F-Conv-3, (**d**) F-TConv-1, (**e**) F-TConv-2, and (**f**) F-Conv-3 of Table S1 (Supplementary Materials).

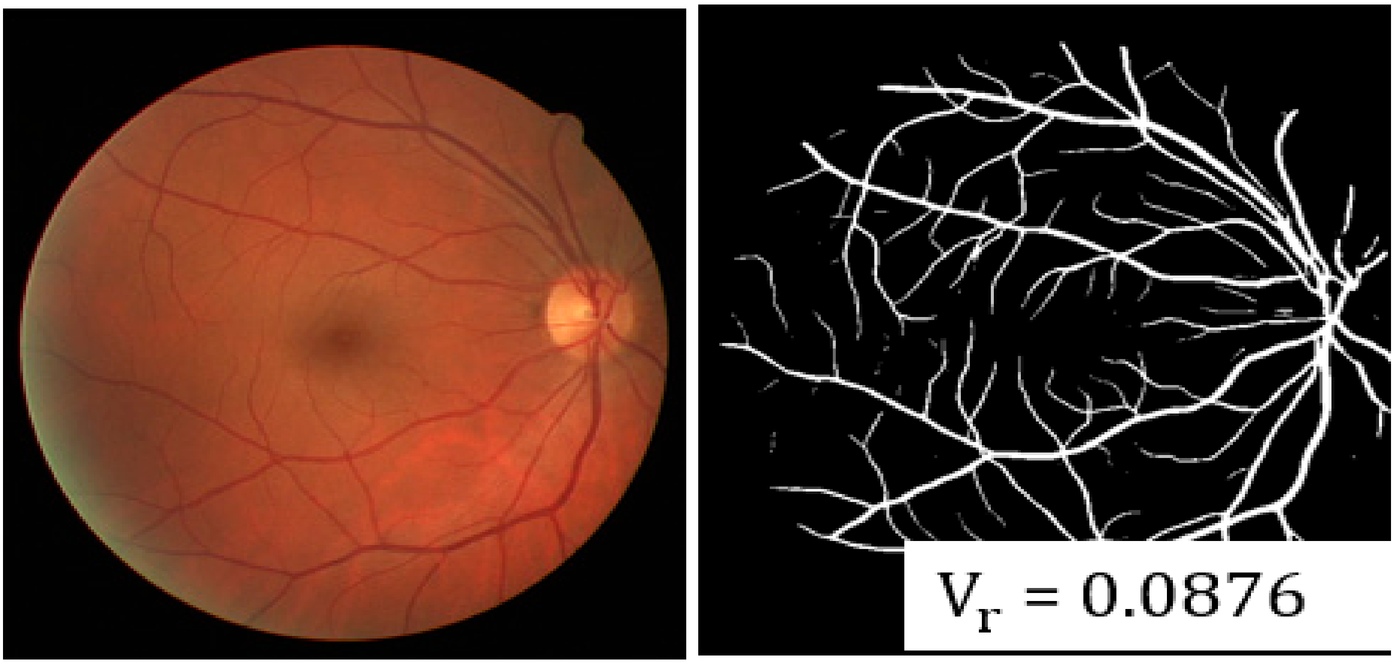


(**a**) (**b**)

**Figure S3.** Sample Images from the DRIVE dataset: (**a**) Original Image and (**b**) predicted mask Image by the proposed method.

$$V_r = \frac{\#\text{ of vessel pixels}}{\#\text{ of background pixels}}. \quad \text{(A)}$$
